# p53 and Zinc: A Malleable Relationship

**DOI:** 10.3389/fmolb.2022.895887

**Published:** 2022-04-13

**Authors:** Jeung-Hoi Ha, Orjola Prela, Darren R. Carpizo, Stewart N. Loh

**Affiliations:** ^1^ Department of Biochemistry and Molecular Biology, State University of New York Upstate Medical University, Syracuse, NY, United States; ^2^ Division of Surgical Oncology, Department of Surgery, Wilmot Cancer Center, University of Rochester, Rochester, NY, United States

**Keywords:** p53 mutants, p53 mutant rescue, cancer, zinc finger transcription factor, zinc homeostasis, zinc metallochaperones, p53 targeted drugs

## Abstract

A large percentage of transcription factors require zinc to bind DNA. In this review, we discuss what makes p53 unique among zinc-dependent transcription factors. The conformation of p53 is unusually malleable: p53 binds zinc extremely tightly when folded, but is intrinsically unstable in the absence of zinc at 37°C. Whether the wild-type protein folds in the cell is largely determined by the concentration of available zinc. Consequently, zinc dysregulation in the cell as well as a large percentage of tumorigenic p53 mutations can cause p53 to lose zinc, misfold, and forfeit its tumor suppressing activity. We highlight p53’s noteworthy biophysical properties that give rise to its malleability and how proper zinc binding can be restored by synthetic metallochaperones to reactivate mutant p53. The activity and mechanism of metallochaperones are compared to those of other mutant p53-targeted drugs with an emphasis on those that have reached the clinical trial stage.

## p^53^, Zinc, and Cancer

Many proteins interact with d-block transition metals such as (in order of decreasing abundance in the human body) iron, zinc, copper, and manganese (Maret, 2016). Transition metal binding serves several purposes: to stabilize protein structure, to facilitate protein-protein interactions, and to provide active centers for enzymatic catalysis and electron transfer reactions. More proteins in the human proteome—about one in ten—are predicted to bind to zinc than to any other transition metal (Andreini et al., 2006). Transcription factors (TFs) are well-represented members of the zinc-binding class in which the metal is used for conformational stabilization. Here, we focus on one protein that exhibits unusual properties among zinc-dependent TFs and plays a pivotal role in cancer: the p53 tumor suppressor.

**FIGURE 1:**
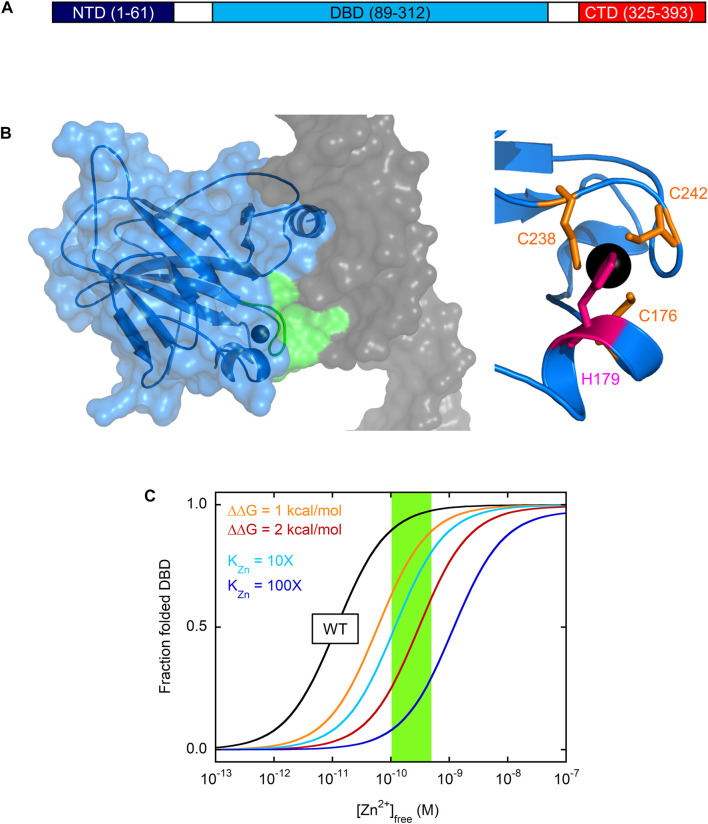
Domain structure of p53 and zinc-dependent folding of DBD **(A)** p53 consists of three functionally distinct domains. CTD encodes for tetramerization and regulatory functions, DBD is responsible for site-specific DNA recognition, and NTD recruits general transcription factors once p53 has bound to DNA **(B)** At left, the X-ray crystal structure of DBD (blue ribbon and surface) reveals that the zinc ion (black sphere) serves to coordinate the L3 loop (green ribbon and surface) in the minor groove of DNA (grey surface). The zinc binding site is shown more closely at right with the zinc-coordinating side chains of Cys and His indicated in orange and pink, respectively. PDB 1TSR **(C)** Zinc drives folding and unfolding of DBD at 37°C. The fraction of folded, active DBD is plotted against [Zn^2+^]_free_ using Eq. 10 from reference (Blanden et al., 2020) and the following values from the same study: K_Zn_ = 1.6 × 10^−15^ M, $\Delta G_{fold}^{apo}=6.9\;\mathrm{kcal}/\mathrm{mol}$ l, K_Zn,U_ = 5 × 10^−8^ M. WT DBD is the black line and the normal range of [Zn^2+^]_free_ in cells is represented by the green box. Mutations that weaken zinc binding by 10-fold and 100-fold are simulated in cyan and blue lines, and mutations that destabilize apoDBD by 1 kcal/mol and 2 kcal/mol are simulated in orange and red lines.

p53 is one of the most intensely studied transcription factors because of its pivotal role in cancer biology. Almost all cancers that progress to clinically relevant tumors do so by inactivating p53 either by mutation or by negatively regulating the WT protein (Levine, 2020). Many of the ways by which p53 functions to suppress tumor formation have been elucidated and include cell cycle arrest, apoptosis, and senescence but newer mechanisms including regulation of metabolic pathways such as ferroptosis have been described (Jiang et al., 2015; Li et al., 2012; Liu and Gu, 2022). It is often noted that p53 is the most frequently mutated protein in cancer. It is less appreciated that tumorigenic mutations are mostly of the missense variety (as opposed to nonsense, deletion, frameshift, gene fusion, and alternate splicing alterations that predominate in other tumor suppressors), localize almost exclusively to its DNA binding domain (DBD; Figure 1A), and are found at nearly every codon position within DBD (Bouaoun et al., 2016; Baugh et al., 2018). The reason for p53s unusual mutational signature is now understood. Missense mutations are selected during tumorigenesis because the mutant proteins impart properties that facilitate tumor progression including enhanced proliferation, migration, and chemoresistance. First described by Levine and colleagues in 1993 (Dittmer et al., 1993), this so-called p53 gain of function (GOF) phenotype comprises a large and ongoing research effort that has been reviewed elsewhere (Bargonetti and Prives, 2019; Amelio and Melino, 2020; Stein et al., 2020). This set of circumstances, together with the biophysical properties of DBD that we discuss below, combine to render p53 particularly susceptible to loss of zinc and DNA binding activity.

p53 is composed of three domains: the N-terminal transactivation domain (NTD), the central DBD, and the C-terminal domain (CTD) that contains the tetramerization and nuclear localization sequences (Figure 1A). DBD is a 24.7 kDa globular protein consisting of a β-sandwich core from which the DNA-contacting α-helix and loops (L1 and L3) project (Figure 1B). The single Zn^2+^ ion, coordinated by C238/C242 in L3 and C176/H179 in another short loop, helps position L3 into the DNA minor groove for consensus sequence recognition. When zinc is removed, zinc-free DBD (apoDBD) remains folded but its thermodynamic stability $\left(\Delta G_{fold}^{apo}\right)$ is diminished. More important to p53 function, however, is that zinc loss perturbs the structure of L3 and surrounding regions, causing apoDBD to lose the ability to differentiate consensus from nonconsensus DNA (Butler and Loh, 2003). Thus, zinc functions to stabilize both the global structure of p53 and that of its local DNA-recognizing elements.

## Malleability of p^53^ Structure

We use the term malleability to describe the acute dependence of p53 structure on the concentration of available zinc ([Zn^2+^]_free_) and on the presence of mutation, such that physiologically-accessible changes in [Zn^2+^]_free_, $\Delta G_{fold}^{apo}$ , or zinc binding affinity can determine whether p53 is folded or unfolded in the cell. Malleability is the product of two opposing properties of DBD: its extreme propensity to unfold, and its high affinity for Zn^2+^ when it is folded.

Fersht and co-workers were the first to observe that zinc-bound DBD (holoDBD) is stable at low temperatures ( $\Delta G_{fold}^{holo}=-9.8\;\mathrm{kcal}/\mathrm{mol}$ at 10°C) but is only marginally stable at body temperature, exhibiting an apparent melting temperature of 42°C (Bullock et al., 1997). We reported shortly thereafter that removing zinc decreases stability substantially ( $\Delta G_{fold}^{apo}=-6.3\;\mathrm{kcal}/\mathrm{mol}$ at 10°C) (Butler and Loh, 2003). Recently, we discovered that DBD is much more unstable than was previously thought at physiological temperature. The folding free energies of all proteins exhibit a parabola-shaped dependence on temperature, the narrowness of which is proportional to the change in heat capacity between the folded and unfolded states (ΔC_p_). ApoDBD possesses an anomalously large ΔC_p_ (7.0 ± 1.7 kcal/mol/K) for a protein its size, which causes $\Delta G_{fold}^{apo}$ to extrapolate to a high, positive value (6.9 kcal/mol) at 37°C (Blanden et al., 2020). The physical basis for apoDBD’s unusually large ΔC_p_ value is not known. The extrapolated stability of apoDBD at 37°C is equivalent to its stability in 4.4 M urea at 10°C and indicates that apoDBD is intrinsically unstable at 37°C.

Intrinsic instability makes the important prediction that [Zn^2+^]_free_ is a primary determinant of whether WT p53 folds or not in the cell. The zinc dissociation constant (K_Zn_) for binding of the metal ion to fully folded apoDBD was measured to be 1.6 × 10^−15^ M at 10°C (Blanden et al., 2020), making apoDBD one of the tightest-binding eukaryotic proteins known (Kochańczyk et al., 2015). Surprisingly, biophysical modeling estimated that instability and high zinc affinity would approximately balance each other such that WT DBD will be on the cusp of unfolding in the cell (Blanden et al., 2020). This point is illustrated in Figure 1C. Zinc induces a folding transition in which the only populated states are unfolded apoDBD and native holoDBD. The midpoint of this transition occurs at [Zn^2+^]_free_ ∼10 pM for WT DBD (black line in Figure 1C), which is near the typical range in cells (100–500 pM; green box) (Krezel and Maret, 2006; Vinkenborg et al., 2009). This transition midpoint corresponds to the apparent zinc dissociation constant (K_Zn,app_) at the conditions of the experiment (37°C in Figure 1C). K_Zn,app_ is identical to K_Zn_ for a protein that’s folded and stable in the absence of zinc, but for an unstable protein some of the binding energy is used to drive folding and K_Zn,app_ is consequently higher than K_Zn_. For these latter proteins, K_Zn_ can only be directly measured under conditions in which the protein is folded (e.g., in the presence of stabilizing agents or low temperature in the case of DBD).

Figure 1C also demonstrates how easily mutations can tip the balance to favor the unfolded state. As one may intuit from this simple modeling, mutations that weaken K_Zn_ by 10-fold (cyan line) and 100-fold (blue line) increase K_Zn,app_ by the same factors. Mutations that do not alter K_Zn_ but destabilize apoDBD by only 1 kcal/mol (orange line) or 2 kcal/mol (red line) require 6-fold and 30-fold higher concentrations of zinc, respectively, to attain the same degree of folding as WT DBD.

## How Malleable Are Other Zinc-dependent Transcription Factors?

The largest class of zinc-dependent TFs are those that employ zinc finger motifs (ZFs) to bind DNA. Zinc finger TFs (ZFTs) and p53 interact with DNA differently. Classic ZFs bind DNA via a short α-helix inserted into the major groove, as opposed to a loop inserted into the minor groove in the case of p53. In addition, a single ZF recognizes three to four base pairs (with a 1-nucleotide overlap between sequential ZFs) (Pabo et al., 2001; Klug, 2010), necessitating multiple ZFs to ensure sequence specificity by spiraling around the major groove. A typical vertebrate ZFT contains 3–15 tandemly-arranged ZFs with which to engage DNA (Klug, 2010; Rutherford et al., 2013). Nevertheless, both ZFTs and p53 use a tetrahedrally-coordinated zinc ion to stabilize their DNA recognition motifs, and ZFTs provide a well-characterized data set for comparing metal affinity and stabilization properties for the same functional class of proteins.

Reported zinc dissociation constants of ZFs vary by ∼7 orders of magnitude, from 10^−7^ M to 10^−14^ M (Kluska et al., 2018a; Padjasek et al., 2020). Several points must be considered, however, when interpreting these data. The first is that nearly all studies of ZFs have employed individual ZF peptides, which are short (∼30 amino acids) and unfolded in the absence of metal regardless of temperature. Thus, $\Delta G_{fold}^{apo}$ and K_Zn_ values are generally not determined and published dissociation constants are K_Zn,app_. K_Zn,app_ alone allows one to calculate metal occupancy as a function of [Zn^2+^]_free_ but not as a function of $\Delta \Delta G_{fold}^{apo}$ that may result from mutation. One exception is the study of the C-terminal ZF from Wilms’ tumor suppressor protein (Chan et al., 2014). The authors reported K_Zn,app_ = 7.5 × 10^−12^ M, $\Delta \Delta G_{fold}^{apo}\leq +2.1\;\mathrm{kcal}/\mathrm{mol}$ , and K_Zn_ = 1.3 × 10^−13^ M. They went on to estimate that several other natural ZFs are unstable by 0–4 kcal/mol but folding is overwhelmingly favored by the ∼−18 kcal/mol free energy change of zinc binding, suggesting that these ZFs are somewhat malleable but not to the same extent as p53.

The second consideration when interpreting zinc binding data is a technical one: an artifact can arise when K_Zn,app_ is measured by adding metal directly to protein and monitoring changes in heat (e.g., isothermal titration calorimetry) or protein spectroscopic properties (UV-visible absorbance, fluorescence, circular dichroism, etc.) (Kluska et al., 2018b). These methods tend to require protein concentrations well above K_Zn,app_, in which case essentially all titrated zinc binds to the protein until saturation is reached. In this regime, stoichiometry can be determined accurately but K_Zn,app_ cannot, resulting in overestimation of K_Zn,app_ (underestimation of zinc binding affinity). Other techniques can circumvent the above issue, such as reverse titration with other transition metals, competition with fluorescent metal chelators, and Zn^2+^ buffering by small molecules (Kluska et al., 2018b). Nevertheless, significantly different K_Zn,app_ figures have been determined for the same ZF peptide, even by the same research group, highlighting the effect of conditions and technique on apparent zinc affinity (Miłoch and Krężel, 2014). Examples from that study include Sp1–3 (K_Zn,app_ = 5 × 10^−9^ M, 1.9 × 10^−13^ M), MTF1-1 (K_Zn,app_ = 2.0 × 10^−10^ M, 2.4 × 10^−12^ M), ZF278-1 (K_Zn,app_ = 6.3 × 10^−11^ M, 9.1 × 10^−14^ M), and ZF133-11 (K_Zn,app_ = 2.2 × 10^−10^ M, 2.8 × 10^−13^ M). The second number (determined by Co^2+^ competition or by using zinc buffers) was the more accurate in all cases.

The last consideration when interpreting K_Zn,app_ data for ZFTs is that potential effects of binding cooperativity are not well characterized. Because ZFTs require at least 3 ZFs to properly engage DNA, ZFs bind with positive cooperativity or at least avidity. The inherent coupling of ZF folding, zinc binding, and DNA binding dictates that zinc ions binding to full-length ZFTs is also likely to exhibit some degree of positive cooperativity. If so, K_Zn,app_ values reported for ZF peptides represent a lower bound of the actual zinc occupancy of ZTFs in the cell.

In summary, ZFs meet one of the criteria for malleability—intrinsic instability in the absence of metal—although not to the same extent as DBD. Malleability also requires that K_Zn,app_ be close to intracellular [Zn^2+^]_free_ (100–500 pM). K_Zn,app_ of most ZFs are in the sub-pM range (Sénèque and Latour, 2010), below that of DBD (K_Zn,app_ ∼10 pM). Thus, available evidence suggests that ZFTs are likely to be fully saturated with zinc under all but the most extreme conditions of metal deprivation. The malleability of p53 appears to be unique among zinc-dependent TFs, although the data are sparse and more studies are needed. Notable exceptions to that assertion include proteins involved in cellular zinc homeostasis, including metallothionein and MTF-1 (the protein that buffers metal concentration and the master transcription factor that coordinates expression of metallothioneins and metal importers/exporters, respectively).

## Classifying p^53^ Missense Mutants

**FIGURE 2:**
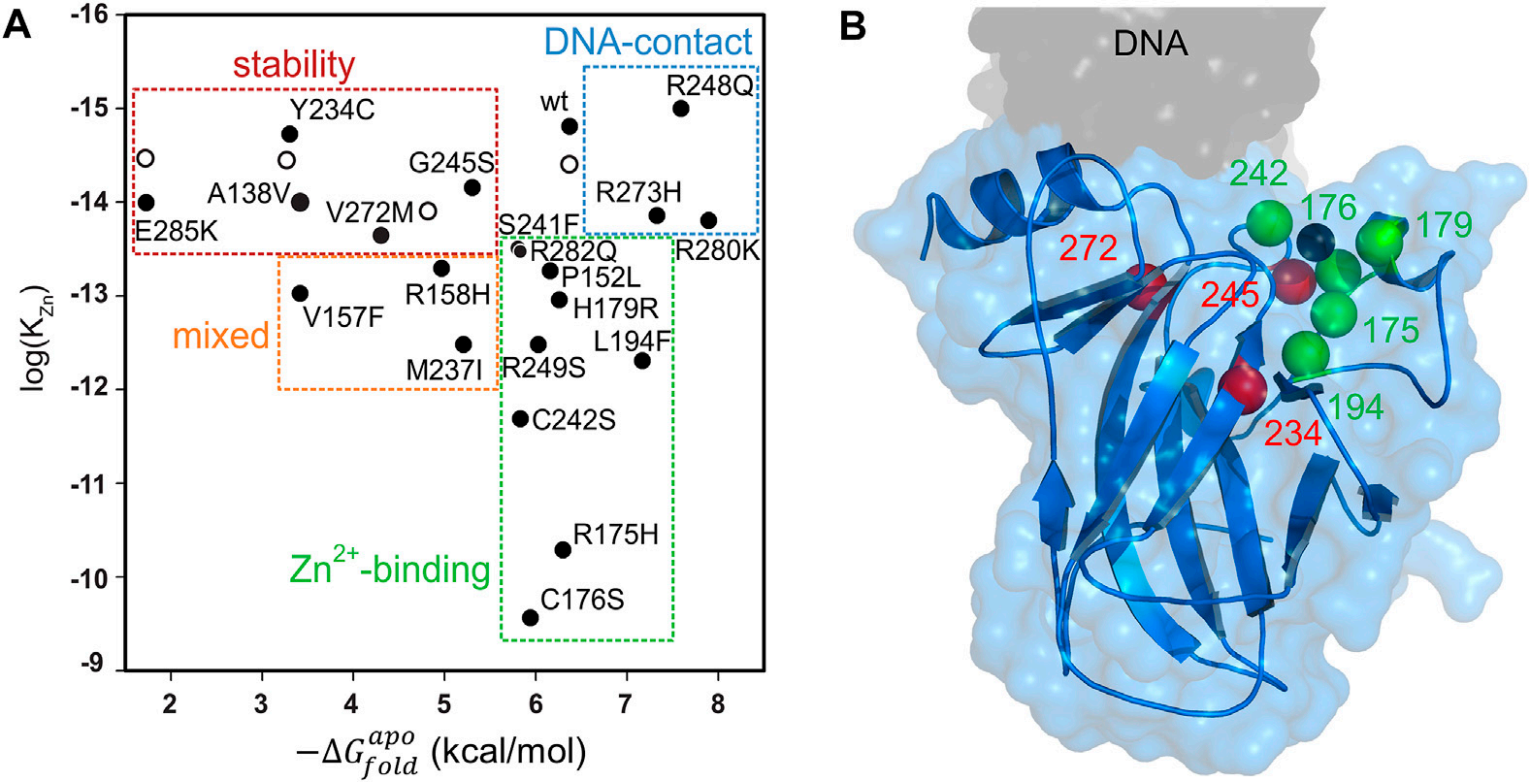
Mutational analysis of DBD **(A)** The top 20 most common p53 mutants can be categorized according to their thermodynamic stabilities (x-axis), zinc binding affinities (y-axis), and DNA binding affinities (determined by separate experiments). Open circles indicate WT DBD in the presence of sub-denaturing concentrations of urea, to illustrate that urea mimics the effect of stability-class mutations. Data are from reference (Blanden et al., 2020) **(B)** Locations of mutations rescued by ZMC1 are shown in the DBD structure. Color coding is the same as in panel **(A)**. The black sphere is the zinc ion. PDB 1TSR.

For some 25 years, numerous research groups have dissected how common tumorigenic mutations affect the structure, function, and folding of p53 [reviewed in (Joerger and Fersht, 2016; Loh, 2020; Bykov et al., 2018)]. These studies have uncovered several foundational mechanisms by which mutations cause p53 to lose its DNA binding activity. *DNA contact* mutations impair DNA binding affinity without weakening K_Zn_ or $\Delta G_{fold}^{apo}$ . *Zinc-binding* mutations compromise K_Zn_ without affecting DNA affinity or $\Delta G_{fold}^{apo}$ . *Stability* mutations diminish $\Delta G_{fold}^{apo}$ without changing affinity for either DNA or zinc. Figure 2A shows how the top 20 most frequently-occurring tumorigenic p53 mutants distribute among these three classes (Blanden et al., 2020). We note that some substitutions affect more than one property (e.g., the mixed class in the orange box impairs zinc binding as well as stability), and other non-DNA contact mutations appear to introduce local conformational changes that propagate to the DNA binding surface, distorting it [e.g., R249S (Bullock et al., 2000)].

The DNA contact class involves substitution of amino acids that directly bind to DNA and these can usually be spotted by inspecting the crystal structure of the DBD/DNA complex. The zinc-binding class is well represented by substitutions near the metal binding pocket (including the zinc coordinating residues C176, H179, C238, and C242), although mutations at more distant positions diminish zinc affinity by unknown mechanisms. The stability class is harder to predict due to the complex origins of protein stability, and members of this category are found throughout the DBD structure.

Although DNA contact, zinc-binding, and stability mutations produce the common result of impairing p53’s ability to bind DNA, it is useful to dichotomize them into these groups for the purpose of developing p53-reactivating drugs. DNA contact mutants are likely to be the most difficult to reactivate, as there is no established strategy for reintroducing lost protein-DNA contacts by small molecules. Stability mutations can in principle be corrected by drugs that bind to the native structure, thereby stabilizing it against unfolding. Examples include the PhiKan family of molecules, which fit into the cavity left by the replacement of the large Tyr side chain with the smaller Cys side chain in the Y220C mutant (Boeckler et al., 2008; Baud et al., 2018; Bauer et al., 2019), and arsenic trioxide, which appears to bind in a natural cavity that exists in WT as well as mutant DBD (Chen et al., 2021). These and other p53-reactivating molecules are discussed in further detail below.

Zinc can be an extraordinarily potent stabilizing ligand, although it is not normally considered as such in the context of drugging proteins. K_Zn_ of WT DBD and stability mutants is orders of magnitude lower than K_d_ of a typical drug/protein interaction, and K_Zn_ of many zinc-binding p53 mutants is still sub-pM (Figure 2A). Thus, there is a great deal of folding free energy to be gained by binding of this 65 Da metal ion. As in conventional drug treatments, Zn^2+^ concentration can be raised to drive binding, and in the case of p53, this is predicted to refold zinc-binding and stability mutants alike. Indeed, elevating intracellular zinc using the synthetic metallochaperone strategy discussed below was found to reactivate five zinc-binding p53 mutants and three stability mutants (Figure 2B) and induce apoptosis in human cells expressing those proteins (Yu et al., 2014a; Blanden et al., 2020).

### Pharmacological Strategies for Reactivating Mutant p53

For over 30 years, the cancer drug development field has recognized that restoring WT structure/function to missense mutant p53 has enormous therapeutic potential. However, only more recently have researchers begun to discover strategies for accomplishing this goal using small molecules or peptides with several drugs now in clinical trials (APR-246, COTI-2, PC14586, and ATO). These are summarized below.

### Prima-1/APR-246

**FIGURE 3:**
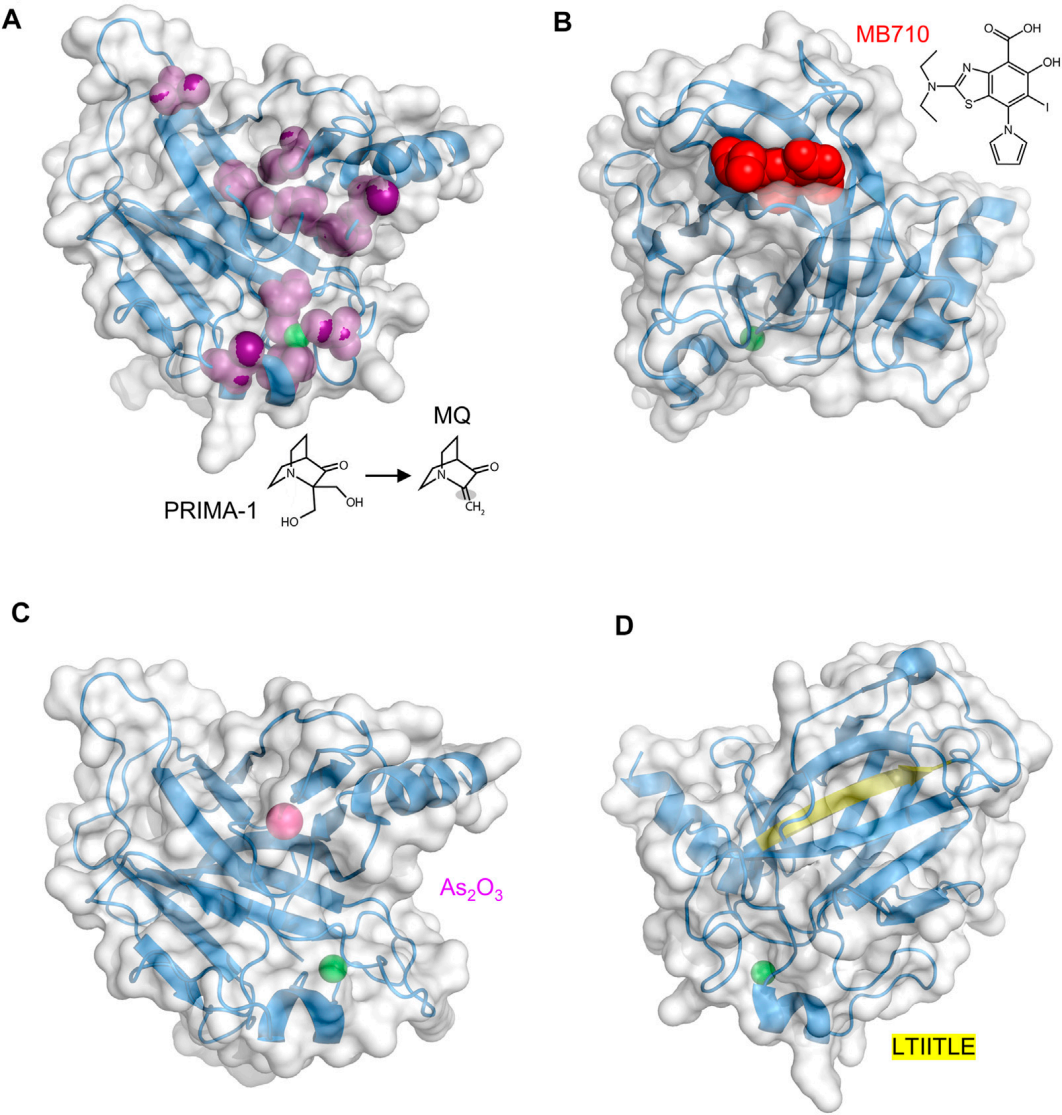
Druggable sites in p53 DBD. The zinc ion is the green sphere in all structures **(A)** PRIMA-1 and its O-methylated analog APR-246 decompose to the active product MQ, which contains a reactive double bond (shaded). MQ forms covalent adducts to a number of Cys residues (purple). PDB 2OCJ **(B)** MB710 binds to Y220C DBD in the crevice left behind by the loss of the Tyr side chain. PDB 5O1I **(C)** The arsenic ion (magenta sphere), originating from ATO, binds in a solvent-inaccessible pocket consisting of C124, C135, C141, and M133. The R249S DBD mutant is shown. PDB 7DHZ **(D)** The buried LTIITLE sequence (yellow) comprises a β-strand that, when DBD unfolds, binds to the same sequence in other unfolded DBD molecules to form the core of p53 amyloid fibrils. PDB 2OCJ.

PRIMA-1 was first reported in 2002 as a compound that could reactivate both conformational and DNA-binding mutants (Bykov et al., 2002). It was discovered by screening a library of small molecules for their ability to induce p53-dependent apoptosis in cancer cells (p53-R273H or p53-R175H). The active species is not PRIMA-1 itself, but its metabolic breakdown product methylene quinuclidinone (MQ) (Figure 3A). MQ forms covalent adducts to multiple Cys residues in DBD, including C124 and C277 (Lambert et al., 2009; Wassman et al., 2013). These covalent modifications thermodynamically stabilize DBD and lead to expression of p53 target genes for proteins such as p21, MDM2, PUMA, and NOXA. Notably, although MQ binds to mutant p53 and increases its transcription activation function, (Nahi et al., 2006), it does the same to WT p53 (Bao et al., 2011), which is generally undesirable.

APR-246, a O-methylated analog of PRIMA-1, was the first in-human mutant p53 reactivating compound to be evaluated in clinical trials (13 to date) (Figure 3A). Phase I and phase II trials (NCT 00900614, NCT02098343, NCT03268382, NCT03072043, NCT03391050, NCT03745716) suggested it was relatively safe and induced apoptosis in tumor cells. This effect, however, was independent of p53 mutational status and was observed in tumors with p53 deletions or with p53-null status (Lehmann et al., 2012; Tessoulin et al., 2014). p53-independent cell killing likely resulted from nonspecific modification of thiol groups in other proteins and small molecules, leading to activation of mitochondrial apoptosis by cytochrome *c* release (Lee et al., 2006), depletion of cellular glutathione (Tessoulin et al., 2014; Liu et al., 2017), and inhibition of thioredoxin and glutaredoxin systems (Peng et al., 2013; Haffo et al., 2018), leading to an overly oxidative state in the cell. These effects are thought to increase therapeutic efficacy especially when combined with other chemotherapeutic agents acting on redox regulation or the unfolded protein response. The recent phase III trial (NCT03745716) that evaluated APR-246 in combination with azacitidine to treat TP53 mutant myelodysplastic syndrome failed to meet its primary endpoint, finding no significant difference between APR-246 plus azacytidine treatment compared to azacitidine alone.

### COTI-2

COTI-2 was the second small molecule with purported mutant p53 rescue activity to undergo phase I clinical trial (NCT02433626). It was discovered using a proprietary machine learning platform (Salim et al., 2016). Interaction with p53 did not appear to be one of the search parameters. COTI-2 induced mutant p53 to refold in cells, as determined by conformation-specific antibodies (Synnott et al., 2018), and it increased expression of p53-dependent genes (Lindemann et al., 2019). COTI-2 is a thiosemicarbazone, like the zinc metallochaperones discussed below, but its mechanism remains undefined. COTI-2 inhibited proliferation of multiple cancer cell lines regardless of their genetic makeup and p53 mutational status, and its mechanisms of action include p53 independent pathways potentially including inhibition of mTOR pathways and AMPK activation (Lindemann et al., 2019).

### Y220C Mutant Stabilizers

The Y220C mutation, found in ∼1.8% of TP53 mutant tumors, is characterized by decreased thermodynamic stability (Blanden et al., 2020; Bullock et al., 2000). The mutation site is distant from the DNA and zinc binding regions of DBD. The crevice that is left behind by replacing the Tyr phenol ring with the smaller Cys thiol group both destabilizes DBD as well as creates a druggable pocket (Boeckler et al., 2008; Liu et al., 2013). In 2008, Fersht and colleagues used an in silico screen to discover PhiKan083 (PK083), a carbazole derivative that bound weakly (K_d_ ∼150 μM) in this pocket and partially restored stability and activity to Y220C DBD (Boeckler et al., 2008). It was reported to not bind WT DBD as determined by NMR experiments. Since then, tighter binding molecules based on iodophenyl (Wilcken et al., 2012) and aminobenzothiazole (Baud et al., 2018) scaffolds have been introduced. MB710 (Figure 3B), an example of the latter class, bound to Y220C DBD with a K_d_ of 4 μM, increased melting temperature by 2°C, and exhibited 7–17-fold lower IC_50_ values against several cancer cell lines, compared to PK083 (Baud et al., 2018). PC14586 is the first orally bioavailable Y220C binder in clinical development that targets a specific p53 mutant. *In vitro* studies showed PC14586 to stabilize the Y220C mutant in cell lines harboring this mutation, resulting in expression of p53 target proteins (p21, MDM2, Bax, and PUMA) and leading to cell cycle arrest (Dumble et al., 2021). In nude mice with p53-Y220C NUGC3 gastric cancer xenograft tumors, oral administration of PC14586 resulted in tumor regression following 3-week treatment. PC14586 is currently undergoing a phase I/II clinical trial in patients with tumors harboring the Y220C mutation (NCT04585750).

### Arsenic Trioxide

In 2018, the FDA approved arsenic trioxide (As_2_O_3_, or ATO) to treat acute promyelocytic leukemia (APL). Like Zn^2+^, the arsenic ion coordinates to thiolate groups of cysteines, and ATO exerts its anti-APL effects by replacing Zn^2+^ with As^3+^ in the RING domain of promyelotic leukemia-retinoic acid receptor alpha (PML-RARα), a protein chimera with oncogenic function (Kaiming et al., 2018). In 2021, Chen et al. reported that ATO rescued multiple p53 hot-spot mutants including R175H, R248Q, R175L, G245S, and R249S (Chen et al., 2021). ATO was identified in a multi-tier screen for compounds that were likely to bind multiple Cys residues. Chen et al. hoped that, like PML-RARα, the arsenic ion would bind to the same residues as Zn^2+^ but with higher affinity, and in the case of p53 increase its thermodynamic stability while allowing it to remain functional. Surprisingly, arsenic did not displace zinc but was instead found to bind in a second, buried pocket in DBD composed of three non-zinc coordinating Cys residues (C124, C135, C141), and M133 (Figure 3C). These findings suggest a mode of action similar to that of PC14586, except applicable to a variety of mutants and not just Y220C. ATO has shown to be synergistic with decitabine *in vitro* (Wu et al., 2017) and a phase I clinical trial is currently underway in which the two agents are being tested in high risk MDS patients with mutant p53 (NCT03855371).

### Inhibitors of p53 Aggregation

ReACp53 is a cell-penetrating peptide that inhibits p53 aggregation. Formation of p53 amyloid fibrils is favored in instances where a mutation such as R248Q or R175H destabilizes DBD, transiently exposing a ‘sticky’ segment (LTIITLE; residues 252–258) that is normally buried (Figure 3D), allowing it to interact with identical segments from other likewise-unfolded DBD molecules (Goldschmidt et al., 2010). The ReACp53 amino acid sequence is almost identical to LTIITLE and it acts by binding to and masking this adhesive segment, preventing the critical nucleation step in p53 amyloidogenesis. When high grade serous ovarian cancer patient-derived primary cells (p53-R248Q) were treated with ReACp53, cytosolic p53 punctate bodies (indicative of protein aggregation) were reduced and nuclear p53 increased, indicative of dis-aggregation and successful refolding (Soragni et al., 2016). Other evidence for reactivated p53 was p53 turnover, cell-cycle arrest, and cancer cell death. Similarly encouraging results were obtained in 3D cell culture, organoids, and mouse xenografts. ReACp53 also restored function to R175H and P223L mutants of p53 in prostate cancer cells (Zhang et al., 2019), consistent with the notion that numerous mutations have the common effect of exposing the LTIITLE sequence. A tripyridylamide-based small molecule (ADH-6) was recently reported to abrogate mutant p53 aggregation and restore its tumor suppressor activity in cell culture and xenograft models (Palanikumar et al., 2021). Oligopyridylamide molecules like ADH-6 inhibit fibrillogenesis of Alzheimer Aβ peptide (Kumar et al., 2018) and islet amyloid polypeptide (Kumar et al., 2015), likely reflecting their interaction with a common core structure found in the amyloid conformation of different proteins.

### Synthetic zinc Metallochaperones: Discovery and Mechanism

**FIGURE 4:**
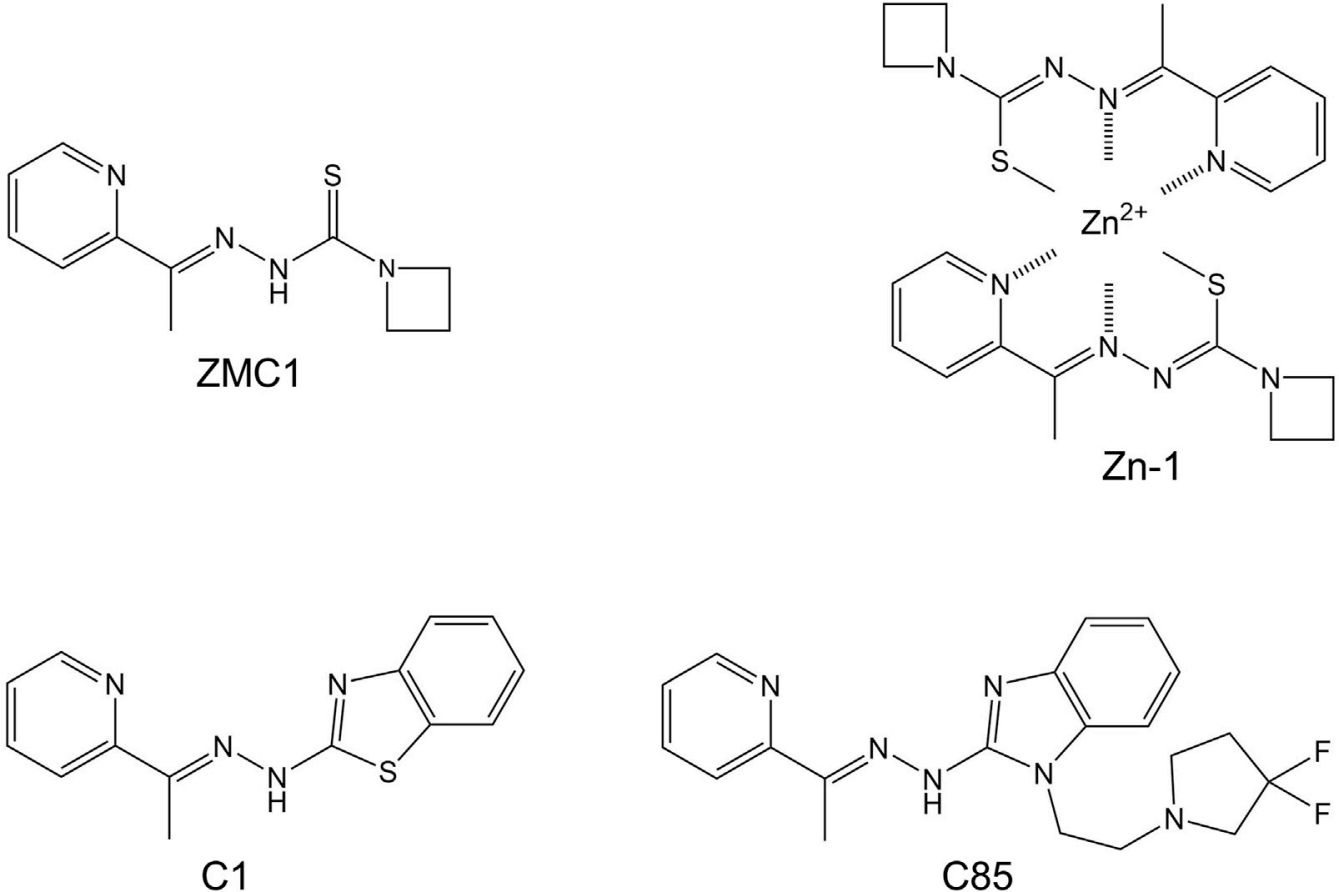
Synthetic zinc metallochaperones based on thiosemicarbazone (ZMC1 and Zn-1), benzothalzolyl hydrazone (C1), and benzimidazolyl hydrazone (C85) cores.

In 2012, zinc metallochaperones (ZMCs) were identified from an in-silico screen of the NCI drug database as reactivators of p53 mutated at codons 175, 248, and 273 (Yu et al., 2012). The highest-scoring compounds belonged to the thiosemicarbazone family with NSC319726 (later named ZMC1) being the most potent (Figure 4). ZMC1 induces apoptosis by restoring WT conformation and transcriptional properties to mutant p53. Because thiosemicarbazones were known to bind transition metals (Yu et al., 2009), and R175H was an established zinc-binding mutation (Butler and Loh, 2003), the mechanism of action was proposed to involve intracellular buffering of available zinc to levels high enough to remetallate mutant p53, but not so high as to induce its misfolding or extensive off-target binding to other proteins (Loh, 2010). This hypothesis was supported by the findings that exogenous ZnCl_2_ and FeSO_4_ enhanced and abolished, respectively, mutant p53-specific cell killing (iron outcompetes zinc for binding to ZMC1). ZMC1 was found to increase levels of a p53-targeted gene (CDKN1A) and inhibit growth of xenograft tumors in mice in a p53-R175H dependent fashion.

**FIGURE 5:**
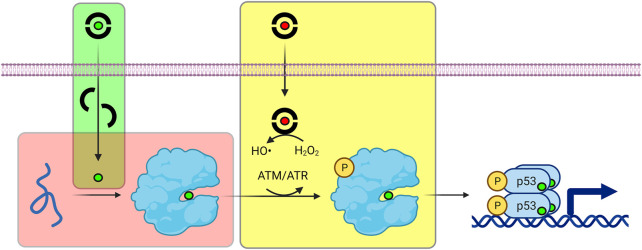
Refolding and activation of p53 by ZMCs. Green box (ionophore activity): ZMCs bind to zinc (green sphere) and copper (red sphere) ions outside of the cell, and the complexes passively diffuse through the plasma membrane. Red box (zinc buffering activity): ZMCs buffer intracellular [Zn^2+^]_free_ at levels sufficiently high to re-metalate unfolded, mutant p53, causing it to spontaneously refold. Yellow box (ROS generating activity): Most ZMCs bind copper much more tightly than zinc. The ZMC-copper complex generates ROS via Fenton chemistry, triggering post-translational modification of p53 (e.g., phosphorylation by ATM/ATR) which facilitates its nuclear entry and transcriptional activation.

ZMCs restore WT structure and function to p53-R175H through a novel mechanism that begins with it binding zinc in the extracellular space in a 2:1 M ratio (green box in Figure 5). Although ZMCs are more effective when administered pre-loaded with zinc (*vide infra*), ZMC1 binds Zn^2+^ ∼100-fold more tightly than does serum albumin and thus ZMC1 can readily obtain metal from the latter source. The albumin/zinc dissociation constant (∼10^−7^ M) (Blindauer et al., 2009; Lu et al., 2008) likely defines the upper limit of K_Zn_ for ZMC efficacy. Both free ZMC and the charge-neutral (ZMC)_2_Zn complex passively diffuse through the cell membrane, with the latter acting as a zinc ionophore. As the intracellular [Zn^2+^]_free_ increases (with ZMC acting as a metal buffer), the metal spontaneously binds to mutant p53, overwhelming its weak binding affinity and restoring proper folding (Blanden et al., 2015; Yu et al., 2014b) (red box in Figure 5). Once p53 is refolded, however, post-translational modifications including phosphorylation, acetylation, and methylation are required for efficient entry into the nucleus (Hafner et al., 2019). In this regard, reactive oxygen species (ROS) generation is the last facet of the ZMC mechanism (yellow box in Figure 5). ROS generation is connected to p53 activation through enzymes that modify p53 as above in response to DNA damage and oxidative stress. Unlike some metal chelators whose binding to iron and copper quenches their redox activity, thiosemicarbazone-iron complexes can efficiently generate ROS through Fenton chemistry (Kalinowski and Richardson, 2007). ZMC1 was shown to bind copper and induce oxidative stress and cell-cycle arrest in glioblastoma cells at picomolar concentration (Shimada et al., 2018). In agreement with ROS generation being important to the ZMC mechanism, ZMC1’s pro-apoptotic activity was reduced by co-treatment with N-acetylcysteine, a reducing agent and free radical scavenger, and was increased by the oxidizing agent diamide (Yu et al., 2012).

Further work demonstrated that the ZMC1-mediated p53 reactivation mechanism is transient over ∼24 h (Yu et al., 2018). Intracellular zinc concentration is tightly controlled by nine exporters (ZnT/SLC30), fourteen importers (ZIP/SLC39) and eleven binding proteins (Andrews, 2001; Palmiter and Huang, 2004; Eide, 2006) whose expression is regulated by metal-responsive element-binding transcription factor-1 (MTF-1) (Maret, 2017). In ovarian cancer cells (TOV112D; p53-R175H) treated with ZMC1, zinc levels peaked at 15 nM by 4–6 h and returned to baseline by 24 h (Yu et al., 2018). This elevated zinc concentration is theoretically sufficient to remetallate p53-R175H (K_Zn,app_ ∼ 2 nM). Despite intracellular zinc decreasing to pre-treatment levels, transcription of zinc exporter and metallothionein genes (ZnT1 and MT1A) remained elevated, and expression of a zinc importer (ZIP10) continued to be downregulated (Yu et al., 2018). In all, ZMC1 treatment altered the expression of 16 of 37 zinc homeostatic genes as determined by RNA Seq analysis. These data indicate that the cell’s zinc homeostatic mechanisms normalize zinc levels and turn off ZMC1, causing mutant p53 to revert to its pre-treatment zinc status. Knocking out MT1A and MT2A expression resulted in earlier, higher, and more sustained zinc levels after ZMC1 compared to control cells. Finally, a single, 30 min exposure of ZMC1 followed by washout reduced the number of TOV112D colonies by ∼50% after 14 day, implying that the relatively brief duration of elevated zinc was sufficient to initiate p53’s irreversible apoptotic programming.

### Zinc Metallochaperones: What’s Next?

A conventional drug is optimized by increasing the binding affinity for its target through chemical modification. ZMCs are different in that the pharmacological agent is not free ZMC or its complex with zinc—neither binds to p53 (Blanden et al., 2015)—but rather the zinc ion itself. The optimal range of ZMC/zinc dissociation constants is already defined and achievable. It is likely bounded by the albumin/zinc interaction at the high end (K_Zn_ ∼10^−7^ M) and by zinc buffering capability at the low end (K_Zn_ ∼10^−10^ M, below which the ZMC would act strictly as a chelator and starve p53 of zinc). The advantage of the zinc ion as a p53 stabilizer is it binds to native p53 orders of magnitude more tightly than most drugs bind to their targets. The disadvantage is that zinc is a promiscuous ion that has the potential to interact with many proteins. Thus, the pathway for bringing ZMCs into the clinic is to minimize off-target toxicity by this and other mechanisms.

One recent advancement was motivated by the X-ray crystal structure of the ZMC1-Zn complex, which was generated by heating ZMC1 and ZnCl_2_ in ethanol with excess triethanolamine (Blanden et al., 2015). Two molecules of ZMC1 coordinated to one Zn^2+^ ion, with enolization of the thiosemicarbazone group resulting in loss of two protons and overall charge neutrality (Figure 4). The pre-synthesized (ZMC1)_2_Zn complex, known as Zn-1, was found to be more effective than ZMC1 in genetically engineered mouse models of breast and pancreatic cancer (Yu et al., 2018; Na et al., 2019). Nonspecific toxicity was also reduced, possibly because the redox-active copper ion was slow to exchange with the pre-bound zinc ion. If the binding and dissociation events between ZMC1, metals, and albumin (the latter being the chief source of available zinc and copper in the blood) were to equilibrate rapidly, then Zn-1 and ZMC1 would be expected to behave identically. That there was a significant difference suggests that the Zn-1 complex persisted long enough to reach the tumor site, and that kinetic considerations may play a role in ZMC efficacy.

Another area of ZMC development is to direct its effects more specifically to tumor sites by separating its activities of p53 refolding and ROS generation. It was established in the mid-1990s that the effectiveness of systemic chemotherapy and radiation (CR) are enhanced in tumors with WT p53 (Lowe et al., 1993; Lowe et al., 1994). As described above, the DNA damage response triggered by CR boosts WT p53 activity (e.g., *via* ATM and ATR kinases). This synergy is one of the theoretical benefits of combining a putative p53 mutant reactivator with CR. Surprisingly, ZMC1 did not synergize with cisplatin, irinotecan, 5-fluorouracil, etoposide, or adriamycin in killing TOV112D cancer cells; only additive or antagonistic effects were observed (Zaman et al., 2019). Similarly, cancer cells were not sensitized to ZMC1 treatment by exposure to ionizing radiation. The reason was proposed to be that the signaling events that CR would normally induce to activate p53 were already being initiated by ZMC1’s ROS generating activity. In agreement, synergy with CR was observed when the free radical scavenger glutathione was introduced, as well as with tool compounds that retained ZMC1-like zinc affinity but bound copper 10^6^–10^7^-fold weaker than ZMC1. These tool compounds were based on a nitrilotriacetic acid scaffold, the poor cell permeability of which precludes their development into drugs. Nevertheless, they illustrate the potential value of developing a new type of ZMC: one with zinc binding properties as ZMC1 but with diminished copper binding that can function as a CR sensitizer for tumors with p53 mutations that can be rescued by ZMCs. This type of ZMC would eliminate the yellow box in Figure 5, instead allowing CR to generate ROS in a more tumor-directed manner.

A series of ZMCs based on benzothiazolyl, benzoxazolyl, and benzimidazolyl hydrazone cores was recently developed (Gilleran et al., 2021; Easmon et al., 1997) with the goals of decreasing the off-target toxicity and multi-target effects (de Siqueira et al., 2019) of thiosemicarbazones while maintaining or increasing potency. The new compounds were designed to retain the 2:1 zinc binding property of thiosemicarbazones (including enolization) as well as their zinc ionophore character. Two lead compounds, C1 (Figure 4) and C2 were found to bind zinc, refold p53-R175H, and kill ovarian cancer cells, all with potencies comparable to those of ZMC1 and with significantly reduced off-target toxicity (Gilleran et al., 2021). A third compound in the series, C85 (Figure 4), exhibited diminished copper binding and functioned as a chemotherapy and radiation sensitizer.

### Consequences of Raising Intracellular Zinc

The pharmacologic mechanism of ZMCs—metal ionophore activity, intracellular zinc buffering, and ROS generation—is reasonably well defined and ZMC design has advanced rapidly. Nevertheless, one challenge that is intrinsic to ZMC therapy remains to be addressed: the need to reduce inappropriate metalation of proteins other than p53. As discussed in the first section of this review, it appears that at least some zinc-dependent transcription factors are already saturated with zinc, so increasing [Zn^2+^]_free_ is not likely to activate their transcriptional functions to the same extent that it activates mutant p53. A recent study employed RNA-Seq to profile global changes in transcription induced by increasing intracellular Zn^2+^ in rat hippocampal neurons (Sanford et al., 2019). Over 900 genes exhibited altered expression. Interestingly, that list includes nine of the top 160 genes activated by p53 in other cell types (*PLK2, CYFIP2, HSPA4L, SLC12A4, FAM212B, ENC1, ARHGEF3, CSF1*, and *CDKN1A*, which encodes for p21, one of p53’s most common transcriptional targets) (Fischer, 2017). These data suggest that elevated zinc causes widespread changes in gene expression in neurons, although zinc signaling is essential to their physiology and neuronal cells may have evolved specialized pathways for regulating and responding to Zn^2+^ fluctuations (Maret, 2017; Aizenman, 2020).

While zinc-induced, non-p53 mediated transcriptional changes are unavoidable, the key to minimizing these effects may be to leverage knowledge of the cell’s zinc homeostatic response in the design and dosing strategy of ZMCs. A single treatment of ZMC followed by washout elevates intracellular zinc for only several hours, but this is long enough for p53 to refold and induce apoptosis. Sustained exposure may not be desirable, as this is likely to elicit a durable zinc muffling response due to increased expression of ZnT and MT proteins. Thus, the pharmacokinetic (PK) profile of ZMCs should be optimized for maximum concentration in the serum (C_max_) followed by rapid clearance. This stands in contrast to the desired PK of most conventional cancer drugs, which is to establish a sustained concentration above EC_50_ for as long as possible, to maximize binding of inhibitor to target. A half-life of 0.5–1 h appears to be optimal for ZMCs.

## Summary

In the context of cancer, p53’s malleability is its downfall. Poised on edge of an energy landscape made precipitous by the opposing forces of intense instability and extreme zinc affinity, any one of hundreds of documented mutations is enough to cause p53 to unfold. Yet p53’s malleability also provides a strategy for its rescue. A primary reason why small-molecule rescue of mutant p53 remains an unmet challenge is that the broad spectrum of mutations found in cancer patients inactivate p53 by multiple mechanisms. Consequently, a drug that reactivates one variant (such as PC14586) may have no effect on another. APR-246, ATO, ReACp53, and ZMCs address this problem by attempting to counter common defects—thermodynamic instability and the structural consequences thereof—that many mutations incur. Singular among these compounds, ZMCs also target the class of mutations that decrease zinc binding affinity, which comprise 12 of the 20 most frequently encountered tumorigenic mutations (Figure 2A).

Zinc has unique advantages and disadvantages as a p53-directed drug. It is well-tolerated and generally beneficial when taken as a supplement. The plasma membrane is impermeable to zinc ions, but it is straightforward to deliver zinc inside the cell using ionophores that are pre-loaded with the metal or abstract zinc from abundant sources in the blood. Finally, zinc binds to folded p53 extraordinarily tightly and therefore possesses a large refolding potential. The major hurdle to zinc therapy is, like that of any drug, to reduce off-target effects. Cellular zinc homeostatic mechanisms are efficient at normalizing zinc levels, which reduces undesired metal binding incurred by ZMC treatment. If zinc homeostatic mechanisms become corrupted in cancer cells, however, it is possible that this may confer sensitivity or resistance (either *de novo* or acquired) to ZMCs. Evidence for dysregulation of zinc transporters for a number of solid organ cancers (breast, pancreatic, and prostate) has been described (Franklin et al., 2005; Kagara et al., 2007; Li et al., 2007; Costello et al., 2012; Costello et al., 2016). Paths forward for bringing ZMCs to the clinic include directing their action more specifically to cancer cells by offloading their ROS generation to CR or employing cancer cell-targeted delivery systems, and to leverage the cell’s own zinc homeostasis mechanisms to transiently elevate intracellular zinc long enough for mutant p53 to begin its transcriptional programming.

## Abbreviations

CR, chemotherapy and radiation; DBD, DNA binding domain of p53; MT, metallothionein; TF, transcription factor; ZF, zinc finger; ZFT, zinc finger transcription factor; ZIP, zinc importing protein; ZMC, synthetic zinc metallochaperone; ZnT, zinc transporter protein.
